# In vivo anti-malarial activity and toxicity studies of triterpenic esters isolated form *Keetia leucantha* and crude extracts

**DOI:** 10.1186/s12936-017-2054-y

**Published:** 2017-10-10

**Authors:** Claire Beaufay, Marie-France Hérent, Joëlle Quetin-Leclercq, Joanne Bero

**Affiliations:** Pharmacognosy Research Group, Louvain Drug Research Institute, Université Catholique de Louvain, Avenue E. Mounier 72, B1.72.03, 1200 Brussels, Belgium

**Keywords:** *Keetia leucantha*, Triterpene esters, Anti-malarial, *Plasmodium*, Oleanolic, Ursolic, Rubiaceae

## Abstract

**Background:**

Considering the need for new anti-malarial drugs, further investigations on *Keetia leucantha* (Rubiaceae), an in vitro antiplasmodial plant traditionally used to treat malaria, were carried out. This paper aimed to assess the in vivo anti-malarial efficacy of *K. leucantha* triterpenic esters previously identified as the most in vitro active components against *Plasmodium falciparum* and their potential toxicity as well as those of anti-malarial extracts.

**Results:**

These eight triterpenic esters and the major antiplasmodial triterpenic acids, ursolic and oleanolic acids, were quantified in the twigs dichloromethane extract by validated HPLC–UV methods. They account for about 19% of this extract (16.9% for acids and 1.8% for esters). These compounds were also identified in trace in the twigs decoction by HPLC-HRMS. Results also showed that extracts and esters did not produce any haemolysis, and were devoid of any acute toxicity at a total cumulative dose of 800 and 150 mg/kg respectively. Moreover, esters given intraperitoneally at 50 mg/kg/day to *Plasmodium berghei*-infected mice showed a very significant (p < 0.01) parasitaemia inhibition (27.8 ± 5.4%) on day 4 post-infection compared to vehicle-treated mice.

**Conclusions:**

These results bring out new information on the safety of *K. leucantha* use and on the identification of anti-malarial compounds from its dichloromethane extract. Its activity can be explained by the presence of triterpenic acids and esters which in vivo activity and safety were demonstrated for the first time.

## Background

Malaria remains an important health problem with 214 million cases reported in 2015 and almost half of the world population at risk [1]. Although malaria control efforts succeed to lower clinical disease incidence by about 40% since 2000, there is an urgent need to discover new anti-malarial drugs, because of parasite resistance and relative low efficacy of vaccines [2, 3]. Nature and mostly plants are a resourceful supplier of potential active metabolites [4] with the successful 2016 Nobel prize example of the sesquiterpenic artemisinin isolated from *Artemisia annua* (Asteraceae) used for thousands years as Chinese herbal remedy [5] or quinine, the first anti-malarial medicine extracted from *Cinchona* bark [6]. Natural compounds present a lot of advantages including their complexity in structure and in mechanisms of action, with potential multi-targeted activity [7]. Moreover, many millions people are relying on traditional medicine for their usual health care according to the World Health Organization (WHO) [8] with over 100 million in Europe and 80 times more traditional healers than medical doctors in Africa. So, plant extracts or mixtures used in traditional medicine and their isolated components, such as triterpenes, play a significant role in the fight against malaria [8, 9].

**Fig. 1:**
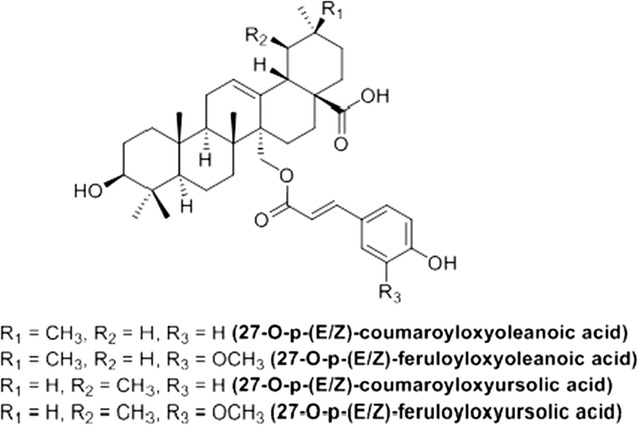
Structure of the eight triterpenic esters isolated from *K. leucantha* (8ETT)

In this context, further investigations were carried out on *Keetia leucantha* (Rubiaceae), a West African tree mainly used traditionally as a whole plant decoction in Benin to treat malaria. Indeed, the dichloromethane extract of twigs exhibits an in vitro selective antiplasmodial activity [10] and reduces significantly parasitaemia in mice infected by *Plasmodium berghei* with 42% of inhibition on day 4 at 200 mg/kg given intraperitoneally. Parasitaemia inhibition of aqueous decoction given by oral route reached 30.8% at the same dose while it was inactive in vitro. Moreover, bioguided fractionations of dichloromethane extract led to the isolation and identification of in vitro antiplasmodial triterpenic acids: ursolic and oleanolic acids (IC_50_ = 32.4 and 59.4 µM, respectively on 3D7 and SI < 3 compared to WI38 mammalian cell line) and eight more selective triterpenic esters (Fig. 1; IC_50_ of the mixture = 1.66 µg/ml, about 2.6 µM on 3D7, SI = 34.6 compared to WI38) [11].

This study aims to obtain specific new information on the composition, the anti-malarial activities and safety of *K. leucantha* twigs and its components to further validate its traditional use to treat malaria. So the amount of ursolic and oleanolic acids was quantified in the crude dichloromethane extract as they were already shown to be effective in vivo [12]. Then, the eight isolated triterpenic esters (referred as 8ETT in this paper), more effective in vitro, were also quantified and analysed for their in vivo anti-malarial activity. Finally, the in vitro haemolytic effect and in vivo acute toxicity of the twigs crude extracts and the 8ETT were evaluated.

## Methods

### Plant material and sample preparation

Twigs of *K. leucantha* (K. Krause) Bridson (Rubiaceae) were collected in Benin (Adjarra, Ouémé) in January 2010 and a voucher specimen was left at the Herbarium of the National Botanic Garden of Belgium (Number BR0000005087129). Two extracts were prepared from powdered twigs, a dichloromethane maceration and an aqueous decoction, as described by Bero et al. [10]. The 8ETT were also obtained by a method adapted from the bioguided-fractionation previously defined [11]. Methanol fraction, obtained after a polyamide column from the crude dichloromethane extract, was submitted to silica gel (Merck 60 0.063–0.2 mm) conventional column (45.0 × 2.5 cm^2^) chromatography. The mobile phase consisted of cyclohexane (Analar NORMAPUR, VWR Chemicals) and ethyl acetate (HyPerSolv CHROMANORM, VWR Chemicals) used in different proportions (v:v) and volumes starting with 4 column volumes (8:2), 12 column volumes (7:3) and about 7 column volumes (6:4), ending elution with pure ethyl acetate then methanol (HyPerSolv CHROMANORM, VWR Chemicals). 6 fractions, pooled according to their TLC profiles, were obtained. Then, those containing the esters mixture were purified on solid reverse phase extraction cartridges (Macherey–Nagel, Chromabond C18 6 ml/1000 mg) with a gradient of methanol–water. Purity was determined by HPLC-high resolution MS (> 95%) and identification confirmed by comparison to reference compounds, as described previously [11].

### Quantification of triterpenic compounds

The identification of ursolic and oleanolic acids in aqueous decoction was performed by HPLC-HRMS [13] and their quantification in the dichloromethane extract by HPLC–UV according to an adapted protocol (Catteau et al., pers. comm.). Briefly, all analyses were performed on a Merck Hitachi LaCHrom Elite HPLC system (L-2130 pump, L-2200 autosampler, L-2300 column oven, L-2450 Diode Array Detector) with a Merck LiChroCART RP-18e column (250 × 4.6 mm^2^, 5 µm). The binary solvent (HPLC grade) isocratic system used involved 80% acetonitrile-methanol (40:35) (HyPerSolv CHROMANORM, VWR Chemicals) and 20% NH_4_H_2_PO_4_ 0.04 M (pH adjusted to 6 with NaOH) at a flow rate of 800 µl/min. All extracts and pure compounds were solubilized in 2% formic acid MeOH and the injection volume was fixed at 20 µl. 7-points calibration curves were realized with reference compounds solutions of both acids (Extrasynthese, HPLC purity ≥ 98%) in a concentrations range from 10 to 200 µg/ml obtained from 0.2 mg/ml stock solutions. Crude dichloromethane extract and aqueous decoction were analysed at a concentration of 1 mg/ml. Tests were achieved two times in duplicate at 210 nm.

The 8ETT were quantified in the dichloromethane extract following the protocol of a validated HPLC–UV method with the same solvent and HPLC UV system on a Phenomenex Luna C18 (2) column (250 × 4.6 mm^2^, 5 µm) (Bero et al., pers. comm.). The calibration standard and dichloromethane extract solutions were prepared at 50 and 2 mg/ml in MeOH, respectively. Tests were achieved in triplicate at 310 nm.

### Animals and parasites

Blood-stage samples of the rodent parasite *P. berghei* NK173 were kept in liquid nitrogen until use. Female Swiss mice (7 weeks of age, 30 ± 4 g) used for efficacy assay as previously described [11] were obtained from Envigo laboratories (Brussels) and female NMRI mice (6–7 weeks of age, 24 ± 3 g) used for acute toxicity according to DNDi drug screening protocol [14] from the house facilities of UCL (Brussels). All in vivo experiments performed during this work were approved by the Ethical Committee for animals use at the Health Sciences Sector of the Catholic University of Louvain (2014/UCL/MD/002).

### Haemolysis test

Haemolysis test was performed following the protocol of Muganga et al. [15] on a 10% (v:v) erythrocytes suspension (buffered with PBS) obtained from fresh O^−^ human blood collected in heparinized-tubes and previously washed with RPMI 1640 medium (Gibco). Triton X-100 at 1% and PBS were used as positive and negative controls and integrity of red blood cells was checked prior to experiment. Crude extracts and pure compounds, prepared in DMSO stock solutions of 10 mg/ml, were tested at 100 µg/ml, corresponding to the highest concentration used earlier in cytotoxic in vitro tests [11], taking into account solvent impact (1% DMSO in PBS, controlled to be not haemolytic) in final results. All samples, in triplicate, were incubated 1 h at ambient temperature with slight stirring (300 rpm). After centrifugation (5 min at 2200 rpm), absorbance (A) of supernatants were read at 550 nm in 96-well microtiter plate to assess haemoglobin release. The haemolysis percentage was calculated as follow:

$${\text{Haemolysis \% }} = \frac{{{\text{A product}} - {\text{A vehicle}}}}{{{\text{A Triton}} - {\text{A PBS}}}} \times 100.$$

### In vivo acute toxicity

The assessment of the highest tolerated dose was based on a DNDi protocol [14]. Crude dichloromethane extract and esters mixture were given intraperitoneally while the oral route was used for decoction. Increasing doses were given to 2 mice every 2 h: 40–60–100–200–400 and 20–30–50–50 mg/kg respectively for crude extracts and pure compounds from stock solutions of 60 and 15 mg/ml. Mice were controlled for any health problem symptoms or behavioural changes and monitored for weight and haematocrit after each injection/gavage and every day during 48 h after administration. If any toxic symptoms are observed, the successive administrations have to be stopped. Moreover, main organs (heart, liver, spleen, stomach, lung and kidney) of mice treated with the 8ETT were observed and weighed wet during autopsy. Control group received the vehicle, distilled water with 10% of tween 80-ethanol (7:3). The total injected dose is finally recorded and this cumulative value will be used to ensure the non-toxicity of in vivo anti-malarial test.

### In vivo anti-malarial activity

Activity test protocol is based on the 4-day suppressive test of Peters recommended by WHO [16]. Mice were randomly divided into 6 mice per group for the 8ETT, 4 for positive control and 5 for the negative one, and were infested intraperitoneally by 4.5 × 10^7^ parasitized erythrocytes. All batches were solubilized in the vehicle previously described (water-tween 80-ethanol) and administered intraperitoneally. The treatment with 8ETT was dosed at 50 mg/kg given four hours after infection on day 0 and repeated during 3 additional days. Methanolic extract of *Cinchona officinalis* containing 5% of quinine (in addition to other related alkaloids) was used as positive control at a 200 mg/kg/day dose corresponding to 10 mg of quinine. Vehicle was used as a negative control. On day 4 post infection, thin blood smears were made from mouse-tail blood and stained with Giemsa. Slides were studied under microscope and parasitaemia was determined by counting at least 1000 erythrocytes. All percentages were normalized according to negative control values and 8ETT percentage was calculated for 80% of responder mice because only mice with parasitaemia counting between 35 and 45% were considered.

### Statistical analysis

Data were analysed by Graphpad Prism6 statistical software and presented as the mean ± standard error of the mean. Differences between independent groups results obtained for in vivo assay were analysed by the non-parametric Mann–Whitney test in one-tailed and by the log-rank test to compare survival curves. Statistical significance between treatments was set at p < 0.05.

## Results

### Quantification of triterpenic compounds

Oleanolic and ursolic acids and 8 ETT, previously identified in the crude dichloromethane extract [11, 17], were quantified in this extract (Table 1). The total of both acids represents 16.9% of this extract, with a majority of ursolic acid (14.2%). These acids were also identified in the aqueous decoction of twigs by HPLC-HRMS in comparison to reference compounds. However, the total quantity remains under the limit of quantification of the UV method and approximates less than 1% according to a semi-quantitative MS method. The amount of triterpenic esters represented 1.8% of the crude dichloromethane extract, about ten times lower than the two acids total percentage. They were also identified in trace in the decoction.

**Table 1:** Percentage of oleanolic and ursolic acids and 8ETT in the crude dichloromethane extract

Triterpenes	%
OA	2.7 ± 0.1
UA	14.2 ± 0.5
Acids total	16.9 ± 0.7
8ETT	1.8 ± 0.3

Mean ± SD made in duplicate

OA, oleanolic acid; UA, ursolic acid; 8ETT, 8 triterpenic esters mixture

### Haemolysis test

The haemolysis capacity of crude extracts and pure compounds mixture was evaluated by measuring haemoglobin release by spectrophotometry (Table 2). Less than 1% of haemolysis was observed at 100 µg/ml compared to a complete one for the positive control. So, these results show the absence of haemolytic activity at this concentration and that the antiplasmodial activities observed in vitro for these natural extracts and components cannot be explained by an indirect toxic effect on erythrocytes.

**Table 2:** Haemolysis percentage

Sample	Haemolysis %
8ETT	− 0.3 ± 0.3
KLD	0.2 ± 0.2
KLA	0.04 ± 0.3
C^+^	99.4 ± 0.1

Mean ± SD made in triplicate

8ETT, 8 triterpenic esters, KLD, dichloromethane maceration of *K. leucantha*, KLA, aqueous decoction of *K. leucantha*, C^+^, detergent

### Evaluation of the in vivo acute toxicity

**Fig. 2:**
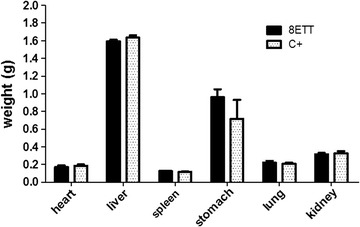
Main organs weight. 8ETT, 8 triterpenic esters; C^+^, vehicle treated control mice

No acute toxic symptom was observed in each group (aqueous and dichloromethane extracts and 8EET) and weight and haematocrit were not impacted compared to controls. Autopsy of 8ETT treated mice did not reveal any macroscopic signs of toxicity and organs weights were normal (Fig. 2). Therefore, the tolerated cumulative doses were evaluated as 800 mg/kg for both crude extracts and as 150 mg/kg for 8ETT. The treatment regimen for efficacy tests can be done at 50% of these highest tolerated doses without any risk of toxicity [14], meaning a maximum of 400 and 75 mg/kg/day respectively.

### Evaluation of the in vivo anti-malarial activity

**Fig. 3:**
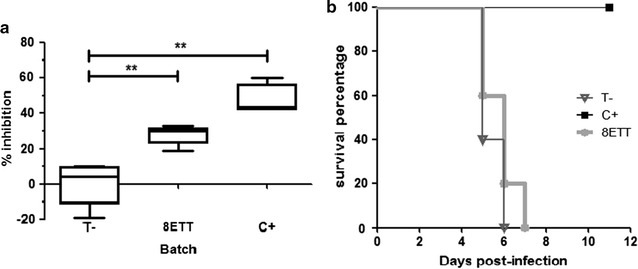
**a** In vivo parasitaemia inhibition on day 4 post-infection in mice infected by *Plasmodium berghei* (** for p < 0.01) and **b** survival. T^−^, vehicle treated mice; 8ETT, 8 triterpenic esters; C^+^, *Cinchona* methanolic extract

The eight triterpenic esters isolated from the twigs of *K. leucantha* given i.p. in mixture at 50 mg/kg/day exhibited an inhibition percentage of 27.8 ± 5.4 on day 4 post-infection. The efficacy difference was very significant (p < 0.01) compared to vehicle-treated mice and the significance value was similar to the one calculated for the *Cinchona* positive control inhibiting parasitaemia of 47.1 ± 8.6% at 200 mg/kg i.p.. For survival analysis, positive control mice survive during all the experiment period and were euthanized after 10 days. Mice in the two other groups did not survive more than 7 days post-infection but a survival increase of 20% at day 5 and 6 was observed for esters treated mice (Fig. 3).

## Discussion

Evaluation of non-toxicity and in vivo activity of natural extracts and pure compounds is essential in the study of medicinal plants and the research of new leads. Pentacyclic triterpenes in therapeutics are often associated with toxicity due to their haemolytic and cytostatic properties [18]. This effect on red blood cells is related to their capacity to incorporate into the lipid bilayer of erythrocytes but also linked to their antiplasmodial activity modifying cells accessibility for parasites [19, 20]. So, as the non-cytotoxicity of crude extracts and their isolated triterpenic esters was already defined on mammalian cells [11], their interactions with red blood cells were evaluated to assess their membrane-damaging capacity. None of them showed haemoglobin release, meaning an absence of haemolytic toxicity. Moreover, ursolic and oleanolic acids were already shown to be devoid of any haemolytic activity [21]. Though, the incorporation into the membrane bilayer and its impacts cannot be excluded and should be investigated, as it is known that ursolic acid and other triterpenes possess membrane disturbing potential [22–24].

It is also crucial to evaluate in vivo toxicity, starting by an acute model, to avoid any side effects during a treatment and to estimate the therapeutic window. The non-acute toxicity of dichloromethane crude extract and decoction completes previous results showing their anti-malarial activity. This evidence on safety in an acute model supports the traditional use of *K. leucantha*, mainly used as whole plant decoction or soup. Then, the absence of acute toxicity for 8ETT was also verified before performing in vivo anti-malarial efficacy tests and settled at a highest tested tolerated dose of 150 mg/kg. The toxicity as well as the anti-malarial effect were analysed on the eight esters mixture because a similar in vitro activity was shown for the mixture and two forms (*cis* and *trans*) combination. Anti-malarial test was performed at 50 mg/kg/day to stay under the 50% calculated highest tolerated dose and because this dose was defined by Nwaka and Hudson for selective promising compounds identification on *Plasmodium* [25] and was two times lower that the one assessed for general antiparasitic compounds [26]. Finally, for this set of tests, the treatment was performed intraperitoneally to compare with previously described extract activity, to limit pharmacokinetics influence and to obtain first efficacy data.

The 8ETT showed moderate anti-malarial activity against *P. berghei* in mice evidenced by the percentage of parasitaemia inhibition. When these results are compared with previous ones [11], it can be observed that the infection was, in this last test, more aggressive with a parasitaemia inhibition about two times lower for the positive control (47.1 vs. 88.3%) and the death of all vehicle-treated mice after less than 7 days. Keeping this fact in mind, the isolated pure compounds were about 1.5 times less active than what was previously observed for the crude extract (27.8 vs. 42.0% inhibition). However, as esters concentration is about 1.8% in the dichloromethane extract, the given dose of 8ETT corresponds approximately to ten times the dose present in the extract. So, even if the eight triterpenic esters have anti-malarial potential, they can only explain a part of the in vivo dichloromethane extract activity previously reported. In addition, ursolic and oleanolic acids, having lower in vitro antiplasmodial activities (IC_50_ = 32.4 and 59.4 µM, respectively, on 3D7) [11] but accounting for about 17% of this extract, can also explain a part of its anti-malarial activity. Indeed, these natural compounds possess many pharmaceutical properties as anti-inflammatory [27–29] and have already shown a significant parasitaemia inhibition in mice infected by *P. berghei* ANKA strain with 97.7 ± 2.6 and 37.4 ± 1.7%, respectively, on day 4 but at a higher dose (200 mg/kg given *per os* daily according to the 4-day suppressive test) [12]. Moreover, in some cases, plant crude extracts can exhibit a higher antiplasmodial efficiency than active isolated pure compounds because of potential addition and/or synergism effects with other compounds, as it is the case for example with artemisinin whose activity is potentiated by some flavonoids present in *Artemisia annua* tea [8, 30, 31].

Literature shows that pentacyclic triterpenes, especially from oleanane, ursane and lupane groups, are known for a wide range of activities among which anti-malarial effects and are largely studied to determine structure–activity relationships [9, 32, 33]. However, only a few of them were tested in vivo. In addition to ursolic and oleanolic acids, maslinic acid increased by more than 80% the mice survival rate after 1 week of treatment at an i.p. dose of 40 mg/kg, showing an arrest of parasite maturation from day 3 to 7 after infection [34]. Asiatic acid, administered p.o. at 10 mg/kg during 5 days starting from 48 h pre-infection suppressed parasitaemia at day 7 (0.13%) and preserved food and water intake of rats. The same protocol applied to chloroquine (30 mg/kg twice daily) did not impact malaria induction (15.72% parasitaemia at day 7) suggesting chemoprophylaxis effects for the triterpenic acid [35]. The chemically synthetized acetyl ester, 3β-acetylursolic acid, exhibited an in vitro activity twice higher than its corresponding acid (IC_50_ = 24.9 ± 7.4 and 52.9 ± 5.7 µM on FcB1 strain and 14 and 36 µM on 3D7 one respectively) [36, 37]. This ester given per os at 400 mg/kg/day also inhibited the parasite development in mice by 94.0% at day 5 post-infection following the Peters test [38]. The 3β-acetylbetulinic acid given to mice by i.p. route caused reduction of parasitaemia (*P. berghei* NK65 strain) of more than 80% on day 4 at 50 mg/kg whereas it was inactive by oral route, potentially due to low absorption and metabolism rate [32]. This may be correlated to the fact that the corresponding acid was inactive at 250 mg/kg i.p. with the same Peters protocol [39]. Two coumaroyl-esters [(E)-*cis* and (Z)-*trans*] of betulin esterified in position 3 were evaluated in vivo at 20 mg/kg/day on *P. berghei* ANKA strain infected mice. Values of parasitaemia chemosuppression were not significant, 16.4 ± 7.6 and 16.5 ± 7.6%, respectively, and efficacy was quite close to that of betulin [40]. These non-significant activities can be due to the absence of carboxylic function in betulin and derivatives. A synthetized hybrid molecule, cinnamic 3β-hydroxyolean-12-en-28-carboxylic anhydride, given i.p. at 50 mg/kg/day during 5 days, reduced parasitaemia by 65.05 ± 4.0% on day 30 in mice infected by *P. berghei* at day 1. For oleanolic acid at the same dose, all mice died after 30 days, showing an important enhancement of anti-malarial activity with a cinnamic acid linked on the carboxylic function [41].

As reported, more in vivo studies are needed to evaluate the anti-malarial potential of pentacyclic triterpenic esters and their derivatives obtained as well as more structure–activity relationships studies to investigate their potency to become leads. Indeed, esterification seems to offer some substantial advantages as shown previously. It clearly strengthens antiplasmodial in vitro activity [11, 33, 36, 37]. As triterpenic acids, mainly lipophilic, exhibit very poor solubility and bioavailability impacting seriously their oral administration, esterification, designed as prodrug esters, should increase solubility in aqueous solution [42], stability in acid stomach environment or during metabolism [43–45] and membrane permeability [46–48] leading to an enhanced bioavailability and thus activity. Moreover, loaded nanoparticles or lipid microparticles should be considered in the evaluation of per os in vivo activity [49, 50].

So, triterpenic esters stability and *per os* efficacy should be investigated taking into account absorption and metabolism impacts to assess their potential to become natural leads for further developments and optimization in the fight against malaria.

## Conclusions

This research attested the anti-malarial in vivo activity of triterpenic esters from *K. leucantha* twigs and their safety in an acute model, as well as for crude dichloromethane extract and aqueous decoction. These esters, as well as ursolic and oleanolic acids, only account for a part of the activity of the crude dichloromethane extract but synergy may also occur between them and different other compounds. This work contributes to the crucial research of new effective anti-malarial compound.
